# Case Report: A literature review of the medical complications arising from etomidate-laced vapes and two case studies of hypokalemia associated with etomidate use

**DOI:** 10.3389/fpsyt.2026.1808879

**Published:** 2026-06-17

**Authors:** Tian Ling Low, Christopher Cheng Soon Cheok, Melvyn Weibin Zhang

**Affiliations:** National Addictions Management Service (NAMS), Institute of Mental Health, Singapore, Singapore

**Keywords:** adrenal insufficiency, case report, etomidate, hypokalemia, substance-related disorders, vaping

## Abstract

Etomidate, traditionally an anesthetic agent, has recently emerged as a substance of misuse through the adulteration of e-vaporizers. Previous reports have raised concerns of associated adrenal dysfunction. In this case report, we describe two patients with a history of etomidate-laced vaporizer use who were incidentally found to have asymptomatic hypokalemia (serum potassium 2.9 mmol/L and 3.3 mmol/L) during outpatient evaluation. Both patients also presented with diffuse and palmer crease hyperpigmentation, a clinical sign frequently seen in adrenal dysfunction. Unlike previously reported cases presenting with neuromuscular weakness or adrenal crisis, this case report highlights subclinical manifestations of endocrine disruption associated with etomidate use. Early screening of asymptomatic hypokalemia in etomidate misuse is therefore important for clinicians treating this emerging group of patients, as this may facilitate timely investigation and prevention progression to severe adrenal complications.

## 1 Introduction

Etomidate is a short-acting, intravenous hypnotic agent widely used for the induction of general anesthesia, rapid sequence intubation, and procedural sedation (1). Pharmacologically, etomidate acts as a positive allosteric modulator of γ-aminobutyric acid (GABA-A) receptors in the central nervous system. While valued in medicine for its relatively stable hemodynamic profile, the drug has recently emerged as a significant public health concern due to its use as a drug of abuse in e-vaporizers, particularly in countries like Singapore, Taiwan and China (2).

Recent public health surveillance and law enforcement data indicate that the non-medical use of etomidate through illicit vaporizer products (colloquially referred to as ‘K-pods’ in Singapore, ‘Space Oil in Hong Kong and ‘Zombie Vapes’ in Taiwan) has escalated sharply across East and Southeast Asia in recent years. In Singapore, from 1 September to 2 November 2025, authorities caught 1,929 persons for e-vaporizer related offences. Of these cases, 167 persons were confirmed to be in possession of e-vaporizers containing etomidate. Random testing of confiscated pods also found that one-third contained etomidate in July 2025 (3). In Hong Kong, e-Cigarette use has been reported to be relatively common in schoolchildren, with surveys reporting that 5.3% of secondary school students have had experience with e-cigarettes (4). From May to December 2024, the Hong Kong Poison Control Centre recorded 45 cases of ‘space oil’ misuse presenting to Hospital Authority emergency departments (5). In Taiwan, authorities have documented dramatic increases in so-called “zombie vape” detections, including a reported surge of over fifty-eight-fold in etomidate-positive samples compared with the previous year, and recreational etomidate use is estimated to account for around 5% of drug cases in recent data (6). These trends underscore the rapid emergence of etomidate-laced vaping products as a substance-use issue with significant enforcement, clinical and policy implications.

Unlike supervised medical administration, the inhalation of etomidate via vapes lacks precise dosing, leading to dangerous neuropsychiatric disturbances and life-threatening medical comorbidities (2). Existing case reports highlight a high prevalence of acute psychiatric symptoms among users, likely triggered by disturbances in GABA-A receptor equilibrium. Documented cases include patients presenting with severe agitation, aggression, mood lability, and suicidal behavior (7, 8). Neurological impairments were common as well, with reports of confusion, insomnia, unsteady gait (ataxia), and drowsiness (8).

The most critical medical concern regarding etomidate misuse is its association with adrenal insufficiency. Etomidate causes a dose-dependent, reversible inhibition of 11β-hydroxylase, a mitochondrial enzyme essential for the synthesis of cortisol and aldosterone (8, 9). While a single clinical bolus dose typically causes transient suppression lasting 6 to 12 hours (1), unregulated inhalation through e-cigarettes involves unpredictable dosing that can lead to prolonged adrenal dysfunction. This biochemical blockade results in the accumulation of precursor hormones like 11-deoxycorticosterone, which exerts mineralocorticoid activity, leading to life-threatening complications such as hypertension, profound hypokalemia, cardiac arrhythmias, and potentially death (10). Case reports from Hong Kong and China have specifically linked etomidate vapes to severe mineralocorticoid excess and hypokalemic paralysis (8, 9), with reports of bilateral adrenal hyperplasia noted on CT scans of chronic etomidate users (9, 10) highlighting the lasting physiological effects of prolonged etomidate use.

In view of the rapid emergence of etomidate-laced vapes and their potential for severe medical harm, Singaporean authorities have adopted a whole-of-government response that integrates legislative control, enforcement, public health surveillance and treatment pathways. From September 2025, etomidate detected in e-vaporizers was brought under enhanced regulatory controls with the reclassification of Etomidate under the Misuse of Drugs Act, allowing offenders to be subject not only to enforcement action but also to mandatory medical assessment and rehabilitation (11). At the clinical level, this means that individuals with a history of using etomidate-laced vapes may be referred to the National Addictions Management Service (NAMS) at the Institute of Mental Health (IMH) as part of a mandatory treatment program which includes both medical and counselling services. Individuals who wish to enroll voluntarily in the program may also do so. A thorough clinical risk assessment based on the DSM-5 Criteria of Substance Use Disorder (12) is conducted, along with basic biochemical investigations (full blood count, renal panel, liver function test) and an ECG to screen for psychiatric and medical complications of etomidate use.

As of 4 May 2026, 256 individuals in Singapore have been referred to NAMS for rehabilitation due to their misuse of etomidate-laced vapes. Our preliminary data sampling of 122 patients shows that approximately 40% (49 out of 122) of patients presenting with history of etomidate use had hypokalemia (defined as serum potassium < 3.5 mmol/L) picked up on screening blood tests, with the large majority of these outpatients being asymptomatic. In this case report, we present two cases of asymptomatic hypokalemia to illustrate its typical presentation in this patient population.

## 2 Case description

**Table 1** compares the two cases described below. Both patients consented to publication of this case report, as stated in the ethics section. This case report was prepared and reported in accordance to the CARE guidelines for case reports.

**Table 1:** Comparison of two case studies.

	Case 1	Case 2
Age	24 years old	23 years old
Gender	Male	Male
Ethnicity	Singaporean Chinese	Singaporean Chinese
Duration of Etomidate Use	3 months	1 year
Frequency of Etomidate Use	2–3 pods a week	2 pods a month
Serum Potassium	2.9 mmol/L	3.3 mmol/L
Blood Pressure	125/80 mmHG	158/98 mmHG
Other clinical signs	Diffuse and palmar hyperpigmentation noted	Diffuse and palmar hyperpigmentation noted

### 2.1 Case 1–serum potassium 2.9 mmol/L

A 24-year-old man of Singaporean Chinese descent with no significant past medical or psychiatric history presented to the NAMS clinic as part of Singapore’s mandatory treatment program for etomidate users. At the time of review, the patient had stopped vaping for two weeks, as he had already been caught and fined by local authorities. He reported a three-month history of using etomidate-laced e-vaporizers socially, averaging two to three e-vaporizer pods each week. He reported a history of one previous episode of vomiting during etomidate intoxication several weeks prior to the consult, as well as nonspecific, intermittent tingling numbness on his upper and lower limbs, but no significant physical symptoms affecting his daily activities. There were no significant psychiatric symptoms (no mood or psychotic symptoms), and no reported withdrawal symptoms after cessation of vaping. While vaping etomidate, he did not report significant psychiatric effects during intoxication but only reported feeling calmer and more relaxed. The patient was diagnosed with Substance Use Disorder (Mild Severity) based on the DSM-5 Criteria.

On physical examination, the patient was noted to have diffuse hyperpigmented skin over sun-exposed areas including the face, forearms and legs. There was notable hyperpigmentation of the palmar creases as well, which is typically seen in adrenal insufficiency and distinguishes the pattern from baseline skin tone. Physical examination was unremarkable otherwise, including a normal neurological examination with no deficits in power or sensation. His vital signs were within normal ranges. Serum potassium was moderately low at 2.9 mmol/L. A routine electrocardiogram (ECG) done found no abnormalities. Routine blood tests including other electrolytes and kidney function, liver function and blood counts were unremarkable.

Alternative causes of hypokalemia were considered as part of the differential but deemed unlikely. Although the patient reported a single episode of vomiting several weeks prior to presentation, this isolated event was unlikely to account for biochemically significant hypokalemia in the absence of ongoing gastrointestinal losses, and the patient continued to tolerate oral intake well without evidence of dehydration or malnutrition. There was no history of poor dietary intake, diarrhea, diuretic use, laxative abuse, or use of medications known to cause renal potassium wasting. In the absence of other identifiable causes, the patient’s hypokalemia was considered to be temporally associated with his recent history of etomidate-laced e-vaporizer use.

### 2.2 Case 2–serum potassium 3.3 mmol/L

A 23-year-old man of Singaporean Chinese descent with a past medical history of well-controlled asthma and no significant psychiatric history presented to the NAMS clinic as part of Singapore’s mandatory treatment program for etomidate users. At the time of review, the patient had stopped vaping for three weeks, as he had already been caught and fined by local authorities. He reported a one-year history of using etomidate-laced e-vaporizers, vaping on average once a month, and using two e-vaporizer pods each time. During intoxication, the patient reported ‘numbing of emotions’ and feeling sedated. There was one previous episode of nausea and vomiting while intoxicated, but no other physical symptoms reported. There were no significant psychiatric symptoms (no mood or psychotic symptoms), and no reported withdrawal symptoms after cessation of vaping. The patient was diagnosed with Substance Use Disorder (Mild Severity) based on the DSM-5 Criteria.

On physical examination, the patient was noted to have diffuse hyperpigmented skin over sun-exposed areas including the face, arms, legs and associated hyperpigmentation of the palmar creases. Physical examination was unremarkable otherwise. He had asymptomatic grade 1 hypertension with a blood pressure of 158/98 mmHg, and otherwise unremarkable vitals. Serum potassium was mildly low at 3.3 mmol/L. A routine ECG done found no abnormalities. Routine blood tests including other electrolytes and kidney function, liver function and blood counts were unremarkable.

Similar to the previous case, there was no history of poor dietary intake, diarrhea, diuretic use, laxative abuse, or use of medications known to cause renal potassium wasting. In the absence of other identifiable causes, the patient’s hypokalemia was considered to be temporally associated with his recent history of etomidate-laced e-vaporizer use.

No acute intervention was initiated for the patient’s asymptomatic grade 1 hypertension, as the patient remained clinically well and there was no evidence of hypertensive urgency or end-organ dysfunction. Given the possibility of transient mineralocorticoid excess related to recent etomidate exposure, conservative outpatient monitoring with serial blood pressure measurements was considered appropriate, with endocrinology referral offered if persistent hypertension or features of adrenal dysfunction emerged.

### 2.3 Management approach

Both the above patients presented with asymptomatic hypokalemia with no significant ECG changes. In line with clinical guidelines for management of hypokalemia (13), both patients were counselled on short term oral supplementation of potassium and advised to increase their dietary intake of potassium. A repeat test for potassium was arranged in one week to ensure correction of hypokalemia. Vitals monitoring is repeated at each subsequent clinical visit to ensure no progression towards severe hypertension or hemodynamic instability. Additionally, patients are offered a referral to endocrinology should there be concerns of adrenal dysfunction or insufficiency.

Both patients were also referred to in-house clinical counsellors as part of the mandatory rehabilitation program, with an aim to maintain abstinence from etomidate-laced e-vaporizers.

## 3 Discussion

The two cases of asymptomatic hypokalemia discussed in our report align closely with current emerging evidence that etomidate misuse is associated with adrenal dysfunction. Existing case reports consistently demonstrate that etomidate misuse is linked to adrenal dysfunction through the inhibition of 11β-hydroxylase, resulting in reduced cortisol synthesis and accumulation of mineralocorticoid precursors with potassium-wasting effects (8–10). Wu et al. (9) illustrated the downstream endocrine consequences of chronic enzyme inhibition, reporting marked elevations of mineralocorticoid precursors associated with hypertension, hypokalemia, and bilateral adrenal hyperplasia, consistent with prolonged adrenocorticotropic hormone (ACTH) stimulation. Chung et al. (8) demonstrated that even intermittent inhalational exposure in adolescents can produce measurable suppression of cortisol synthesis, highlighting the sensitivity of the adrenal axis to inhaled etomidate. At the severe end of the spectrum, Qin et al. (10) described progression to overt adrenal insufficiency and adrenal crisis, reflecting failure of compensatory mechanisms in the setting of sustained steroidogenic blockade.

While previous reports have highlighted severe inpatient presentations such as hypokalemic paralysis (9) and adrenal crisis (10), our report highlights outpatient cases of asymptomatic hypokalemia which may precede more severe endocrine or systemic complications. A finding of asymptomatic hypokalemia may represent an early and subclinical manifestation of etomidate-induced adrenal dysregulation and may go easily undetected if not screened for. Therefore, clinicians managing patients with a history of etomidate misuse should remain vigilant for potential asymptomatic hypokalemia and proactively monitor for signs and biochemical indicators of adrenal dysfunction.

Moving forward, we would suggest that the management of patients with a history of etomidate misuse presenting in the primary care or outpatient setting to include, at a minimum, assessment of vital signs and a renal panel (comprising of serum sodium, potassium, creatinine and urea) looking for hypokalemia. This facilitates early detection of asymptomatic hypertension and hypokalemia, both of which result from the mineralocorticoid excess seen in etomidate use and may be easily picked up through routine screening. When detected early, these abnormalities can often be monitored and managed in the outpatient setting; however, more severe derangements may warrant prompt referral to endocrinology or the emergency department for intravenous potassium replacement or treatment of hypertensive emergencies. In addition, symptoms such as generalized fatigue and weakness, anorexia, nausea and vomiting, weight loss and postural hypotension may signal progression of adrenal insufficiency and should prompt early referral to endocrinology. Although severe adrenal crisis is unlikely to present in outpatient settings, clinicians should remain vigilant for features such as severe hypotension, shock, and altered mental status, which require immediate emergency management.

Notably, although neither patient in our report exhibited overt physical complications of adrenal dysfunction, both demonstrated hyperpigmentation, a finding commonly observed in adrenal insufficiency. Reduced cortisol synthesis diminishes negative feedback on the pituitary, resulting in elevated ACTH levels. This in turn promotes hyperpigmentation via increased binding of ACTH and α-melanocyte-stimulating hormone to melanocortin 1 receptors (14). Such cutaneous hyperpigmentation is a characteristic feature and diagnostic clue for Addison disease and may similarly indicate underlying adrenal dysfunction in individuals using etomidate-laced e-vaporizers. Accordingly, the presence of cutaneous hyperpigmentation may aid in raising clinical suspicion for etomidate misuse in patients who present with unexplained hypokalemia accompanied with behavioral changes and may prompt clinicians to include etomidate as part of the toxicology screen.

Our report highlights the need for further research to better characterize the medical consequences of etomidate misuse. Specifically, more studies are required to determine how the duration and pattern of etomidate abuse correlate with changes in serum potassium and adrenal function. Clinicians, particularly psychiatrists managing patients with a history of using etomidate-laced e-vaporizers, should be aware of the potential endocrine and metabolic complications, even in the absence of overt symptoms, and should proactively look out for signs of adrenal dysfunction and screen for hypokalemia. Additionally, interdisciplinary collaboration with our endocrinology colleagues is essential in developing evidence-based clinical guidelines to screen, monitor, and manage individuals exposed to etomidate, ensuring early detection and prevention of potentially serious endocrine complications.

### 3.1 Limitations

A number of limitations should be acknowledged in this case report. Firstly, exposure to etomidate was based primarily on patient self-report, and no confirmatory toxicology testing was performed at the point of assessment. However, both patients had previously been apprehended by the Central Narcotics Bureau in possession of etomidate-laced e-vaporizers and had admitted to etomidate use, which increases confidence in the exposure history. Nevertheless, no urine toxicology screening was conducted during follow-up to objectively confirm abstinence after cessation of vaping.

Second, we did not obtain biochemical markers of adrenal dysfunction, including serum cortisol, ACTH, aldosterone, or renin levels. As such, the proposed mechanistic association between etomidate misuse, 11β-hydroxylase inhibition, and the observed hypokalemia and hyperpigmentation remains inferential rather than directly demonstrated in our patients. Nonetheless, this proposed mechanism is supported by previously published case reports from other institutions documenting biochemical evidence of adrenal suppression and mineralocorticoid excess in individuals using etomidate-laced vapes.

Third, follow-up data were limited, as both patients defaulted repeat potassium testing, and subsequent endocrine evaluations were performed at external institutions that were inaccessible to our team. This reflects an inherent service limitation within the National Addictions Management Services (NAMS), which functions as a tertiary addiction psychiatry service rather than a restructured hospital with integrated endocrinology support; consequently, endocrine investigations and specialist management must be referred externally according to institutional protocol.

In addition, the quantity of etomidate exposure could not be quantified, as the concentration of etomidate within illicit vape pods is neither standardized nor disclosed. This precluded meaningful dose-response analysis and limits comparisons across cases and studies.

Despite these limitations, the present report aims to highlight the potential clinical value of routine screening for hypokalemia and adrenal dysfunction in individuals with a reported history of etomidate misuse, even in the absence of overt symptoms.

## Data Availability

The original contributions presented in the study are included in the article/supplementary material. Further inquiries can be directed to the corresponding author.
